# Risk Response for Municipal Solid Waste Crisis Using Ontology-Based Reasoning

**DOI:** 10.3390/ijerph17093312

**Published:** 2020-05-09

**Authors:** Qing Yang, Chen Zuo, Xingxing Liu, Zhichao Yang, Hui Zhou

**Affiliations:** 1 School of Safety Science and Emergency Management, School of Management, Wuhan University of Technology, Wuhan 430070, China; yangq@whut.edu.cn; 2 School of Management, Wuhan University of Technology, Wuhan 430070, China; 247821@whut.edu.cn (C.Z.); zhouhui246683@whut.edu.cn (H.Z.); 3 College of Information and Computer Sciences, University of Massachusetts Amherst, Amherst 01002, MA, USA

**Keywords:** risk response, municipal solid waste, ontology, transformation, rule reasoning

## Abstract

Many cities in the world are besieged by municipal solid waste (MSW). MSW not only pollutes the ecological environment but can even induce a series of public safety crises. Risk response for MSW needs novel changes. This paper innovatively adopts the ideas and methods of semantic web ontology to build an ontology-based reasoning system for MSW risk response. Through the integration of crisis information and case resources in the field of MSW, combined with the reasoning ability of Semantic Web Rule Language (SWRL), a system of rule reasoning for risk transformation is constructed. Knowledge extraction and integration of MSW risk response can effectively excavate semantic correlation of crisis information along with key transformation points in the process of crisis evolution through rule reasoning. The results show that rule reasoning of transformation can effectively improve intelligent decision-making regarding MSW risk response.

## 1. Introduction

Rapid increase in population, urbanization, and economic development has led to significant increase in the production of municipal solid waste (MSW) annually [1]. About 4.3 billion urban residents will generate 1.42 kg/capita/day of MSW by 2025 [2]. In 2004, China surpassed the US as the world largest waste generator. It was predicted that in 2030, China will likely produce twice as much MSW as the US [3].

MSW generation has soared in recent years resulting in overloading of waste management facilities and incapacity of waste management departments to deal with the volume of MSW generated, especially in the developing countries [4]. The improper disposal of MSW will not only cause a series of ecological and environmental crises that pollute the air, water and soil, but also cause public crises, e.g., social security and public health [5]. A waste explosion in Manila in July 2000 killed more than 100 people and injured thousands [6]. A sensational “smelly” campaign broke out in Lebanon in July 2015, and the public’s protest campaign against pollution in the waste dump finally turned into a political event [7]. In the past, there have been dozens of mass incidents involving the MSW crisis in China. The MSW crisis affects almost every aspect of social life [8]. In addition to exploring the issue of waste management from the perspective of technology and environmental protection, we should also pay attention to the public crisis caused by the MSW problem, and actively seek ways to respond to the MSW crisis and reduce economic losses and casualties [9].

The research on the MSW crisis is mainly carried out from two aspects: MSW management and public crisis management. MSW management mainly focuses on its technology, management system, collection and transportation from the perspectives of science and engineering, environmental influence, and law. Public crisis management mainly focuses on the mechanism and causes of crisis evolution [10]. Most perspectives use qualitative and common methods such as the three-stage model [11], 4R model [12] and five-stage crisis management model [13] based on life cycle theory to study the concept, characteristics, classification, causes, and countermeasures of crisis events; and theoretical models such as probability theory [14], system dynamics and game theory [15] to analyze their evolutionary laws [16]. Current crisis response system lacks an exploration of the public crisis caused by MSW, as well as the specific methods and models for the optimization of a path towards crisis transformation. The research mode comes to be narrowed, while empirical and case analysis become less common. The exploration and discovery of the key risk points or potential opportunity points in the crisis evolution path are insufficient [17].

Public crises caused by MSW often have inadequate auspices, with high complexity and potential secondary hazards [18]. Such crises are severely destructive and have a wide range of impacts, requiring the government to make critical decisions under a time limit and high uncertainty [19]. The response management for the crisis in China is at the stage of data collection and information fusion, in which the following problems exist: (1) weak monitoring and early warning capabilities for crisis information [20]; (2) crisis information is widely distributed in different institutions without sufficient integration and sharing [21]; (3) crisis information has poor semantic relevance; the granularity of relative structured knowledge is coarse and the knowledge system lacks ontological support [22]; (4) there is still a big gap in the current crisis response system for effectively expressing an extensive, detailed, comprehensive, and deep-level knowledge correlation among diverse sources of information; knowledge discovery, semantic reasoning, knowledge conversion and value-added approaches are still difficult [23]; (5) the key risk points or potential opportunity points in the crisis evolution process are not sufficiently tapped to support the government’s decision-making [24].

In recent years, as an important formal representation method, ontology has obtained extensive attention and in-depth application in related fields such as knowledge engineering, natural language processing, artificial intelligence, and semantic web [25]. Ontology models constructed by integrating reusable domain knowledge can be used in intelligent decision-making for risk response [26]. Using the Semantic Web rule language (SWRL) to expand ontology’s reasoning ability can make full use of expert experience, cases and other unstructured texts to complete knowledge organization, intelligent retrieval and information reasoning in risk response systems [27].

Although the ontology model has been applied in the above-mentioned fields, there are few applications in the field of MSW crisis [28]. Since MSW processing in China is still dominated by the government, the related industries that dispose MSW cannot rely on the market to operate, so policy interference is more volatile. The analysis of the MSW crisis is mostly qualitative, mainly based on logical expression and ideological discussion. The method we propose can apply ontology modeling to condense these logical systems and ideas into knowledge rules and integrate various logical systems and ideas through objective reasoning which is more systematic and scientific.

In order to reduce the huge negative effect of the MSW crisis on urban modernization and eliminate the negative impact of information asymmetry and poor knowledge exchange on the risk response system, this study innovatively uses ontology reasoning technology to construct a MSW risk response model. Through case analysis and reasoning, this research can extract previous successful cases and failure experiences, integrate massive data and complex information, explore key risk transformation points, and form a decision support system. Therefore, this study might be useful for waste management authorities to respond to MSW risks.

## 2. Research Framework

In the preparedness phase of risk response, due to the diversity, uncertainty, and special factors in the dynamic evolution of the MSW crisis, it is hard to generate an effective MSW risk response strategy plan quickly by a simple statistical model and expert evaluation method [29]. The method of constructing an MSW risk response model based on ontology inference technology proposed in this paper aims to use the efficient information utilization of ontology technology to integrate case resources and other information relating to the MSW crisis. It can provide decision makers with an exploration of key risk transformation points when handling similar MSW crisis events and generate suggestions about decisions to assist in risk response. This method is appropriate when the waste management authorities need to use historical case resources to generate decisions for MSW risk response [30]. Consequently, ontology-based reasoning is employed to support MSW risk response decision-making including ontology-based case representation, SWRL-based rule reasoning and a case study in Z city.

(1) Ontology-based case representation

**Figure 1:**
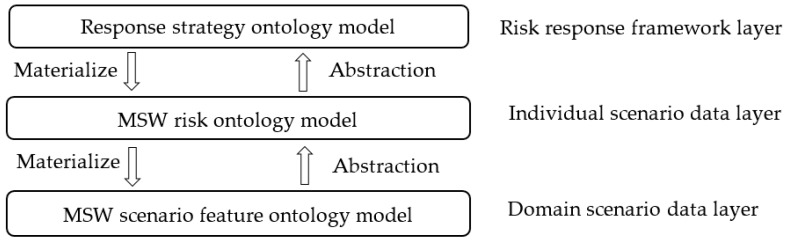
Municipal solid waste (MSW) crisis ontology model construction framework.

An ontology-based case representation method is illustrated to represent MSW scenario features, MSW risks, and response strategies involved in the MSW crisis case, which describes the concept hierarchy of MSW scenario features, MSW risks and semantic relations among the elements of response strategies [31]. This MSW scenario ontology model is the domain scenario data layer. The MSW risk ontology model is the individual scenario data layer. The response strategy ontology model is the risk response framework layer. Between the risk response framework layer and the domain scenario data layer is the process of data abstraction, which in turn is the process of abstraction. The relationship between the three ontology layers is shown in Figure 1:

(2) SWRL-based rule reasoning

A SWRL-based rule reasoning method is proposed by refining rules with logical connotations from identifiable data structures in the risk response knowledge domain [32], and then matching inference rules with existing ontology knowledge to explore new risk transformation points. 

The first step is to extract rules from the existing risk response experience texts as the basis for subsequent reasoning [33]. 

The second step is to sort out the hierarchical relationship of rules by using the logical analysis ability of the decision tree. 

**Figure 2:**
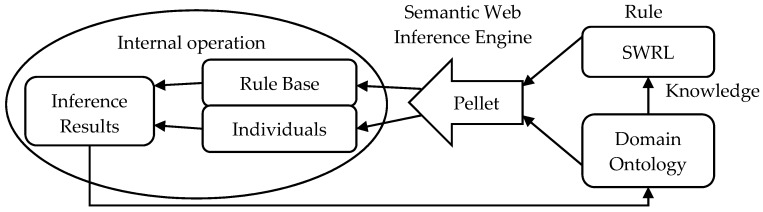
Reasoning system framework.

**Figure 3:**
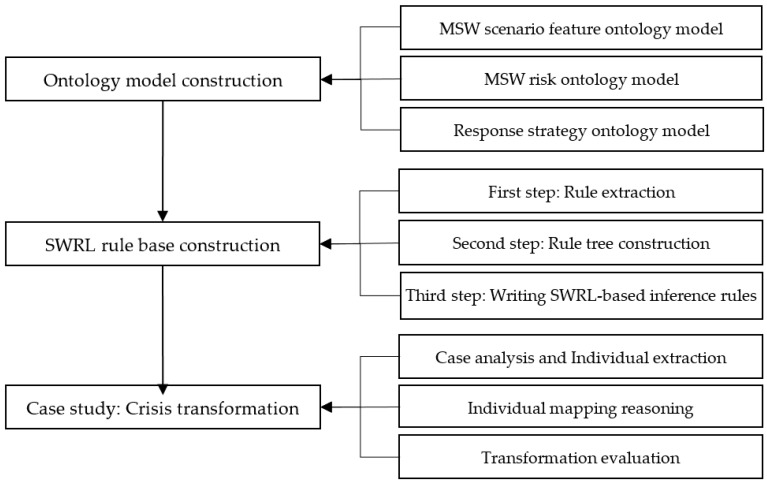
Research framework of MSW risk response based on ontology.

Building the SWRL-based inference rules is the third step. The operation of the inference system combines the domain ontology containing knowledge of SWRL rules and imports it into the Pellet inference engine built into Protégé (a free, open-source ontology editor and framework for building intelligent systems, https://protege.stanford.edu/) for inference [34]. Inside the inference engine, the actual objects of the rule base are diverse individuals added to the ontology [35]. Finally, Protégé will add the inferred new attributes and relationships to the original ontology to enrich the knowledge system in the domain [36]. The framework of the reasoning system is shown in Figure 2:

In conclusion, the research framework of this paper is shown in Figure 3:

## 3. Ontology-Based Case Representation

### 3.1. Case Structure

The MSW risk case structure can be organized as: MSW risk case = < MSW scenario feature (M), MSW risk (R), response strategy (S) > [37]. Specifically, MSW scenario ontological features are descriptions of fundamental features such as natural and social attributes of MSW. MSW risk ontology is a description of various types of crisis events caused by MSW, including environmental events and public crisis. Among them, the type “Not In My Back Yard” (NIMBY) event is particularly significant [38]. Response strategy is generally in the form of tasks toward overcoming MSW risk. The related tasks mainly refer to government actions, residents’ responses and intervention from relevant participants.

### 3.2. Ontology Modeling Elements

The term “ontology” is defined as “an explicit specification of a conceptualization” [39]. In previous studies [40,41,42,43], knowledge of case ontologies is formalized using five types of component as ontology modeling elements: concepts, relations, functions, axioms and instances. In this section, we build MSW risk case ontology that is denoted as MRCOntology. Functions and axioms can be regarded as the specific relations. Therefore, MRCOntology can be expressed as:

MRCOntology = < MRCOConcepts MRCORelations MRCOInstances >

(1) Concepts: In MSW risk cases, concepts are used to describe major elements of the MSW scenario, MSW risks and response strategies.
(2) Relations: Relations represent the semantic relationships between concepts including “isA”, “hasStrategy”, “hasResult”, “isPartOf”, “use”, “hasAction”, “imposeOn”, “isInstanceOf”, etc. Most of them are binary relations denoted as: MRCORelation = R (MRCOConcept, MRCOConcept’).
(3) Instances: Instances are the specific individuals in the case base.

### 3.3. MSW Risk Case Ontology Model

The ontology can be divided into four types [44,45]: top-level ontology, domain ontology, task ontology and application ontology. The MSW crisis case ontology model system constructed in this paper corresponds to four types of ontology. The top-level ontology defines all the conceptual elements in the system, such as actuality, abstraction, temporality, event, etc. [46]. Domain ontology outlines MSW scenario features and MSW risks, and task ontology illuminates MSW risk response strategies. The entire MSW case ontology system is integrated into an application ontology. Next, the detailed process of constructing MSW scenario feature ontology, MSW risk ontology and response strategy ontology using Protégé software will be described [47,48].

#### 3.3.1. MSW Scenario Feature Ontology Model

**Figure 4:**
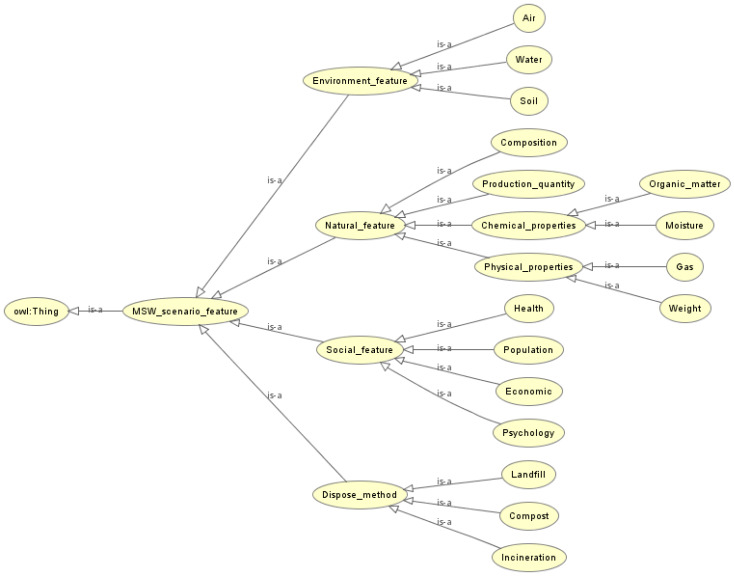
MSW scenario feature ontology model.

As shown in Figure 4, the MSW scenario feature ontology model has four branches: (1) Natural Features. The natural features with great impact on the MSW crisis are mainly production quantity, composition, physical and chemical properties [49]. (2) Disposal Method. Disposal method for MSW often has direct influence on the possible crisis events. At present, the general disposal methods of MSW include landfill, incineration and composting [50]. (3) Environmental Features. Environmental features indicate the adverse effects that MSW may have on the surrounding environment, such as air pollution, water pollution, and soil pollution. (4) Social Features. The social features of the MSW crisis scenario are mainly the population, economy, health and psychology affected by the MSW crisis [51], which plays an essential role in MSW risk response strategy planning. It is worth mentioning that we only give the main MSW scenario features in Figure 4.

#### 3.3.2. MSW Risk Ontology Model

**Figure 5:**
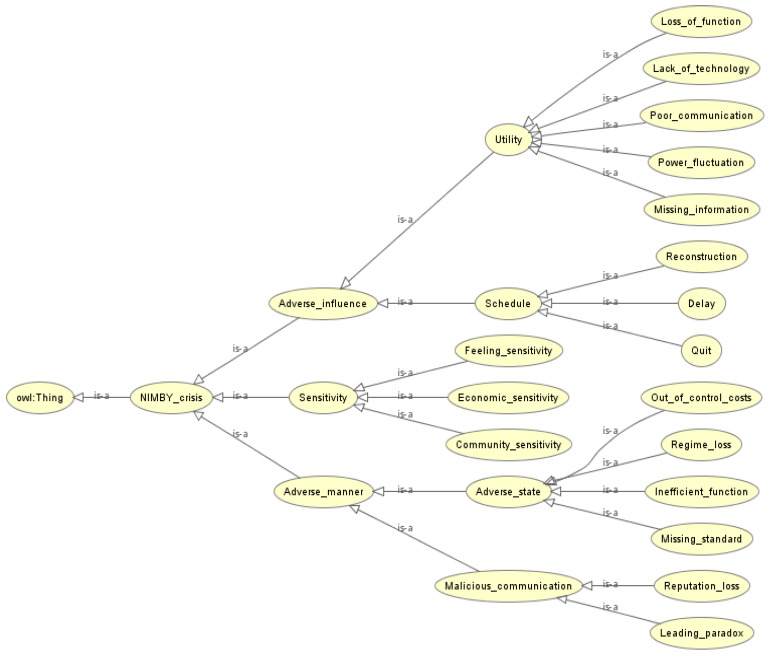
MSW risk ontology model.

The current typical MSW crisis events are the NIMBY events and derived events caused by waste incineration [52], so this section takes the scenario of the NIMBY crisis as an example to build the MSW risk ontology model. Due to analogous combinations of factors, similar crisis scenarios tend to produce the same risk situations [53]. Therefore, historical cases of MSW risk response can provide effective support to reduce the possibility of the occurrence of NIMBY crisis, which can be denoted as replication principle. In this situation, the MSW risk ontology model has three branches (see Figure 5):

(1) The adverse influence of the NIMBY crisis is generally recognized as the failure result of the evolution of the MSW crisis, which mainly includes two aspects: utility reservation and schedule execution. Utility reservation usually manifests as loss of function, lack of technology, poor communication, power fluctuation or missing information. The schedule execution mainly indicates whether the waste incineration project would ultimately be reconstructed, delayed or quit.
(2) The risk sensitivity of MSW includes the residents’ sentiment sensitivity, community sensitivity, and social, economic and environmental sensitivity. Clarifying the sensitivity of each factor is conducive to a positive risk response [54].
(3) An adverse manner can increase the probability of MSW crisis including diverse adverse states such as missing standard, regime loss and inefficient function, leading to paradox along with reputation loss caused by malicious communication.

#### 3.3.3. Response Strategy Ontology Model

**Figure 6:**
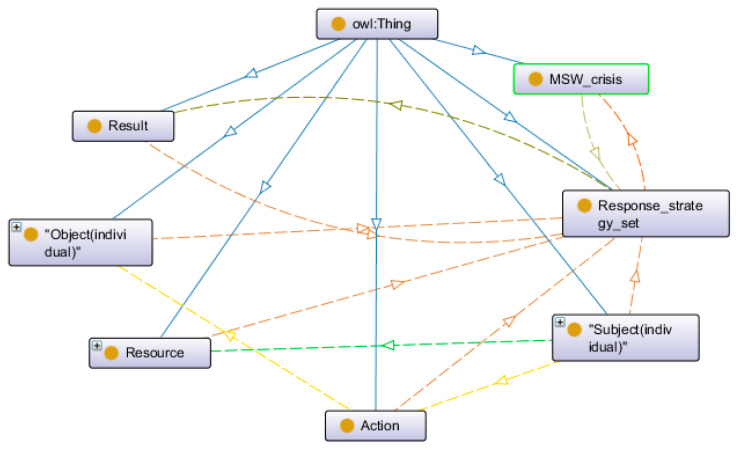
The conceptual model of response strategy ontology.

Other than static domain knowledge, MSW risk cases also contain a lot of procedural knowledge in the form of response strategy. The MSW risk response strategy consists of multiple response strategies, and each response strategy corresponds to different types of crisis events. Moreover, each response strategy consists of four elements: subject, object, resource and action. For instance, government (subject) needs to establish (action) a negotiation channel (object) with compensation requirements (resource). As for the evolution of the MSW crisis in China, the main participants are regional hierarchy governments and residents [55]. Response strategy can be expressed by ontology to provide a general problem-solving model. Figure 6 shows the conceptual model of response strategy ontology within a clockwise logical chain. The main composition of the strategy set can be described as follows: the subject uses resources to take action on the object, and this action will cause the strategy set to produce results.

There are many class-to-class relationships in the strategy response ontology model (as presented in Table 1), and these relationships make risk response strategies closely linked. The result of the response strategy is summarized as mitigation, avoidance or transfer of MSW risk [56,57].

## 4. SWRL Rule Base Construction

### 4.1. Rule Extraction

Various strategies such as environmental assessment, public participation, and economic compensation have been adopted to resolve conflicts in the MSW crisis, but the performances often are not significant [58]. Although most of China’s NIMBY conflicts are processed through conventional approaches, some circumstances of innovation appear, such as promoting legal construction and social cognition, that are very similar to “conflict transformation” [59]. The idea of “conflict transformation” believes that the transformative power contained in the event should be valued, and conflicts should be transformed into opportunities for achieving better balance [60]. Governance of conflicts should not only take “elimination of conflicts” as the ultimate target, but also promote a constructive response to conflicts in the process of thereby changing relations, interests, and situations [61]. Conflict transformation proposes novel requirements for the current risk response system of MSW. 

How to capture the transformative force in time when the conflict happens, transform the conflict into an opportunity, and provide the most accurate and effective reference for the next step of strategy formulation, have become profound issues. The risk response for the MSW crisis based on ontology reasoning is an exploration of conflict transformation. Integration of knowledge ontology and SWRL reasoning ability helps provide feasible suggestions rapidly for conflict transformation in the MSW crisis.

#### 4.1.1. Text Interpretation

The establishment of SWRL rules is based on the interpretation of several excellent articles in the ideological field of “conflict transformation” of MSW [62,63]. The influence of external factors such as “knowledge” and “resources” on the overall evolution and development of the crisis is analyzed at first. Then, we extract the inference rules that are conducive to the analysis of the conflict transformation and complete the expansion and update of the existing MSW risk response ontology. The rule extraction for NIMBY conflict transformation can be carried out by following three aspects: status alteration, strategy upgrade and event conversion.

(1) Status alteration: Taking several typical NIMBY crisis events as case studies [64,65,66], at first the residents are in a status of strong resistance, which may trigger a series of mass incidents. However, with the popularization of scientific knowledge, the residents gradually realize that confronted with severe MSW problems, blindly opposing the establishment of a waste incineration plant is not the best choice. Here, we suppose that after residents participate in the NIMBY conflict, they choose to learn relevant professional knowledge about MSW problems. Knowledge here generally refers to legal knowledge, news reports, professional technology, environmental awareness, and information identification, so that their identity will be changed from ordinary residents to expert citizens with professional knowledge. According to the abstract syntax of SWRL rules, if residents increase their professional knowledge, then residents are expert citizens. Then, this rule is expressed in SWRL as follows:
  Resident(?x)^Knowledge(?z)^hasIncrease(?x,?z)->ProfessionalCitizens(?x)
  here ?x and ?z represent individuals. ^ means co-occurrence relation. -> mean transformation reasoning.

(2) Strategy upgrade: The strategy upgrade focuses on the method transformation, mainly about the shift from fierce and peripheral resistance to internal communication channels and negotiable atmosphere [67,68]. At this stage, some domestic NIMBY events gradually reflect the upgrade of methods and strategies. People pursue rights protection via diverse soft routes, such as hearing reconsideration, environmental impact assessment litigation, and actively promoting negotiations. The formation of such new strategies is inseparable from the resources such as organizational ability, resource mobilization ability, and action ability. If residents with diverse professional knowledge are organized with economic and material support, they have the strength to negotiate with the government or companies. Then it can be envisaged that disordered professional residents have been upgraded to a complete organization. This constitutes a rule at the second level. Residents increase their organizational capacity as the body of rules, and residents upgrade to a complete organization as the head of rules. Then, this rule is expressed in SWRL language as follows:
  Resident(?x)^Knowledge(?z)^hasIncrease(?x,?z)^Resources(?y) ^hasAdd(?x, ?y) -> CompleteOrganization(?x)

(3) Event conversion: The transformation of conflict matters from unitary to pluralistic, competitive to constructive is directly affected by the requirements of residents or organizations. Residents have shifted from fierce struggle to milder strategies such as “invited discussions”, “asking for hearings”, and “written proposals”, reflecting the changes in defending their own interests [69]. With the increase of relevant knowledge and the addition of resources, residents are gradually aware of the root causes of the risk from waste incineration, so the requirements imposed on the government are more rational and comprehensive. It can be recognized that if the residents with enhanced knowledge have made reasonable demands to the government in the form of a unique organization, the government would accelerate the reform of environmental protection policies during the negotiation. This constitutes the end rule that residents are directly related to the outside world following conflict transformation. Then, this rule is expressed in SWRL as follows:
  Laws(?a) ^ Government(?q) ^ Resident(?x) ^ Knowledge(?y) ^ Resources(?z) ^ Requirement(?p) ^ hasIncrease(?x, ?y) ^ hasAdd(?x, ?z) ^ hasProduce(?x, ?p) -> hasEstablish(?q, ?a)

Above mentioned examples for each aspect of conflict transformation are just examples from real scenes.

#### 4.1.2. Rule Tree Construction

**Figure 7:**
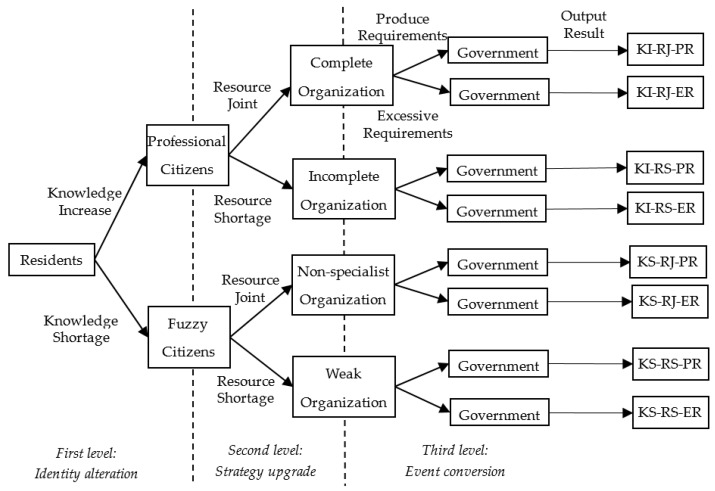
The rule tree based on hierarchical theory.

In the logical construction of the inference rules, a tree structure can help sort out the effects of different triggering factors on the transformation of the NIMBY crisis at each level. Combined with the explanation of the inference rule extraction process in previous sections, an inference rule tree based on the risk response ontology of the MSW crisis is constructed, as shown in Figure 7.

The top node of the tree is one of the participants in the NIMBY crisis, that is, the core object of this reasoning study, i.e., residents. 

Starting from the top node, the first level describes the reasoning process of the transformation of resident status. It takes the “knowledge” as trigger conditions, considering two test conditions of “knowledge increase” and “knowledge shortage”, and outputs residents’ status as “professional citizens” or “fuzzy citizens”. For example, after the residents increase their “knowledge” of environmental protection awareness, their status will be transformed into “professional citizens”. 

The second level describes the reasoning process of the strategic upgrade after the transformation of resident status. It takes the various “resources” as trigger conditions, considering two test conditions of “resource joint or “resource shortage”. The residents of different status are exported as “complete organization”, “incomplete organization”, “non-specialist organization” and “weak organizations”. For example, if the “professional citizens” increase the resources of organizational capacity, they will be upgraded to a “complete organization”. 

The third level describes the reasoning process of event conversion after the upgrade of the strategy and tests the residents who join different organizations with the triggering conditions of “produce requirements” and “excessive requirements”. The third level is the final level of the inference rule tree, so the output is the system’s inference results. In order to evaluate the degree of crisis transformation of the output results, this article uses a combination of abbreviations to mark each output path. For example, “KI-RJ-PR” represents the path: “Knowledge Increase” -->” Resource Joint” -->”Produce Requirements”.

### 4.2. SWRL Semantic Principles

The implementation of SWRL formal semantics and inference support is usually to map the ontology language to a known logical system and display it according to ontology Wed Language (OWL) classes, attributes, individuals, and data values [32]. Its basic form is a deduction representing premises and conclusions. Both premises and conclusions can include single or multiple basic propositions, and the basic propositions are logically AND [70]. The rules and specific formal expressions of SWRL described are as follows:

Rule = B1˄B2˄B3˄B4˄...... Bi ....... ˄Bi -> A.

where 1 ≤ i ≤ n. In SWRL rules, variables need to be prefixed with “?” to identify them. ˄ means Logical AND. When it represents deductive rules, it can be explained that if {Bi} are all true, then A is also true [28].

### 4.3. Building SWRL-Based Inference Rules

After elaborating the method of extracting the inference rules of the MSW risk response ontology and using the rule tree to sort out the logic construction process of the rule base, the SWRL rule base can be constructed by combining the SWRL semantic principles. For each branch in the rule tree, the internal nodes can be sorted into the head of the rule, and the trigger conditions can be sorted into the body of the rule. 

For example, in the first level of the rule tree, when the residents lack knowledge, their identities will be transformed into fuzzy citizens. The residents lack of knowledge is the body of the rule, and the fuzzy citizens are the head of the rule. The rules are described in SWRL as: Resident(?x) ^ Knowledge(?z) ^ hasLackOf(?x, ?z) -> FuzzyCitizens(?x);

In the second level, when resources are added to the fuzzy citizen group, the group will be upgraded to non-professional organization. The fuzzy citizen added resources are the body of the rule, while the non-professional organization is the head of the rule. The rules are described in SWRL as: FuzzyCitizens(?x) ^ Resources(?y) ^ hasAdd(?x, ?y) -> Non-specialistOrganization(?x).

Since the third level is the end output part of the reasoning system, it connects the results of a variety of triggering conditions from the previous levels. According to the different types of “knowledge” and “resources” added, a variety of different risk conversion results could be originated from the same branch. For example, after residents have increased their knowledge of law and raised their social status, they may urge the government to reform related policies. Similarly, after residents actively grasp the relevant professional knowledge about waste incineration, with the addition of organizational capacity and resources, civil organizations may request negotiation demands to the government, which promotes the establishment of mutual acceptable means of communication. In the first two levels of the rule tree, the above two rules are “knowledge addition” and “resource addition”. Due to the difference of specific trigger factors, two conflict conversion results are generated, so two rules can be written: 

Laws(?a) ^ Government(?q) ^ Resident(?x) ^ Knowledge(?y) ^ Resources(?z) ^ Requirement(?p) ^ hasIncrease(?x, ?y) ^ hasAdd(?x, ?z) ^ hasProduce(?x, ?p) -> hasEstablish(?q, ?a) and Result(?a) ^ Government(?q) ^ Resident(?x) ^ Knowledge(?y) ^ Resources(?z) ^ Requirement(?p) ^ hasLackOf(?x, ?y) ^ hasAdd(?x, ?z) ^ hasProduce(?x, ?p) -> hasEstablish(?q, NegotiationChannel).

Therefore, the writing of rules at the third level is different from the first and second levels. Impact difference underlies the decision-making limitations of rule tree with only eight output points under the third level structure, and also provides more logic output for rule base.

**Figure 8:**
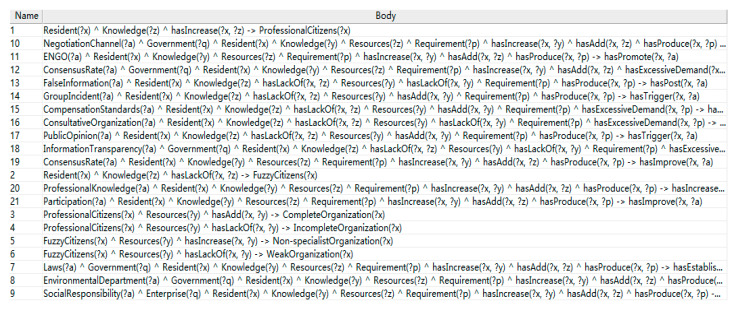
The rule base in Protégé.

In summary, based on the inference rule tree, the SWRL rule base containing 22 inference rules for MSW risk response ontology is established using the rule extraction method introduced in Section 4.1, as shown in Table 2. The rule base ensures that each branch of the rule tree has at least one risk transformation output, and there can be multiple output results on the branch containing more transformation factors. The rule base established for SWRL can also be adjusted specifically to practical applications. The rendering of the rule base in Protégé5.3 is shown in Figure 8:

## 5. Case Study: Reasoning Based on SWRL Rules

The proposed risk response ontology model based on the MSW crisis can assist in analyzing transformative power in crisis events, and then help formulate the subsequent risk response plan. The constructed SWRL rule base expands the reasoning ability of the ontology and provides a more accurate reference to judge the direction of risk conversion. Therefore, this section will demonstrate the proposed analysis method through specific case study and clarify the specific operational process of the risk response ontology for the MSW crisis.

### 5.1. Case Selection and Analysis

The selected case needs to meet the dual requirements of “grounded theory” and “multi-case study” [71,72]. For the former condition, theoretical sampling should be used, i.e., the number of cases can be small or even only one, but it should be a phenomenon with great research potential. In the latter condition, it is necessary to ensure that the sample follows the “rule of replication” and either produces the same result (item-by-item) or makes changes based on predictable causes. 

In summary, the following sample selection criteria are set [73]. First is typicality and representativeness. The case realized an incredibly significant transformation of conflict. In the meanwhile, the time, location and groups involved were different, ensuring its representativeness and coverage. Second is observability and accessibility. The transformation process and effect can be clearly observed and the cases have extensive public attention, especially academically. Information asymmetry and missing information could then be avoided.

Based on extensive literature concerning typical NIMBY cases of China’s waste incineration in the past ten years, an incident in Z city was selected as the target case [64,65,66]. A waste incineration facility in Z City went through several stages: direct start-up, public protest, outbreak of conflict, cessation of construction, rectification and upgrading, and reconstruction on original site. Finally, the project was successfully implemented. The government’s lack of information disclosure in the early stage of the NIMBY incident led to the accumulation of negative energy and dissatisfaction. After being protested against by the public, the government intended to gain public understanding by issuing announcements and holding press conferences, but ignored the real demands of the public, causing the crisis to worsen. Facing the overwhelming reaction from the public, Z city government continued to compromise and terminated the project construction temporarily. In the end, however, the project team and government adhered to an open attitude, and vigorously promoted public understanding and supervision of the operation process of the project. Eventually, these decisions and actions won trust and support from the public, and the project was restarted and put into trial operation two years later. Surrounding residents gained environmental improvement in their living area and full respect. In turn, they consciously maintained the environment and public health. That is to say, the incident achieved crisis transformation.

The entire evolutionary development process of the NIMBY event in Z city contains many transformative forces that affect the subsequent evolution, which is consistent with the transformational thinking of the risk response ontology for the MSW crisis [74]. Due to the large scale and large number of participants, the event has representativeness as required. The impact of the incident has also attracted widespread attention in the environmental protection industry, and rich academic discussion. In summary, the incident in Z city satisfies the selection criteria of case analysis and is suitable as the object of the reasoning experiment for the risk response ontology MSW crisis.

### 5.2. Individual Extraction

The situation status data of crisis events should be imported into the rule base in the form of ontology individuals to realize the inference expression of crisis events [75]. Therefore, after selecting the NIMBY event in Z city as the object of case analysis, it is necessary to instantiate the scenario of the crisis event evolution process including extracting the concept individual, adding corresponding attributes, and establishing the connection among individual, class, and attribute [76]. In this way, the SRWL rule base can make inferences based on various attributes of the individual, and analyze the risk conversion direction and degree of each individual [77].

Describing a case scenario marked by time notes could help figure out the evolution process clearly and find out similar cases for learning from experience [78]. The NIMBY event in Z city lasted for 22 months. The whole process included 17 prominent time points. Non-critical time points with weak correlation are screened out and 10 nodes are retained to show the crisis transformation process [79]. The description of these 10 time nodes and scenarios is shown in Table A1 (Appendix A).

**Figure 9:**
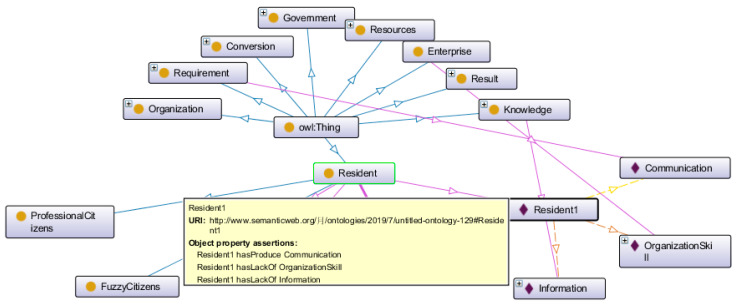
The ontoGraf of Resident1 in Protégé.

Take the time node on 21 June 2016 as an example to introduce the individual 1 extraction process. In this scenario, the local residents participated in the incident through various channels without sufficient and clear information. It can be considered that the residents in this scenario did not have the attribute of “knowledge increase” and no identity transformation. Then, the residents participated in discussion of the issues when they received uncertain information through the major network platforms. In this case, the residents are split as independent individuals. Without any resources, they have to get help from public opinion and the media. It can be considered that the residents in this scenario did not have the attribute of “resource adding”, and no strategy upgrade has occurred. After analyzing the various attributes of the residents, the extracted information can be compiled in the form of ontology Wed Language (OWL) into an individual that Protégé can identify and analyze. The individuals in the scenario are named with orders like “Resident1, Resident2, Resident3” and the object attributes of Resident1 are: “hasLackOf” information, “hasLackOf” organization skill, and “hasProduce” communication. Resident1’s attributes and connections are shown in Protégé as Figure 9.

According to the information expressed by the scenario at each time node, the individuals and attributes are extracted, and then compiled into a Protégé readable individual using the OWL paradigm. By processing the selected 10 time nodes in the same way, 10 individual objects can be obtained using SWRL rule base reasoning analysis. After the scenario processing is completed, the individual extraction shown in Table A2 (Appendix B) will be obtained.

### 5.3. Individual Mapping Reasoning

**Figure 10:**
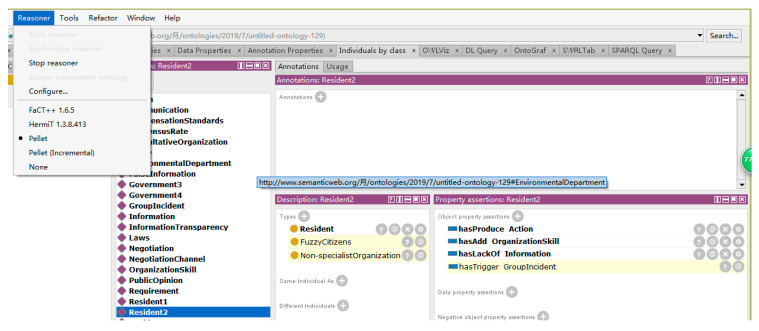
The inference result of individual in Protégé.

The experimental process based on rule inference first establishes 10 scenario individuals drawn from the Z city case in Protégé, and sets the corresponding Object property for each individual, and then starts the Pellet inference engine to get the corresponding inference results. As shown in Figure 10, the dark shaded box in the figure represents the result of reasoning.

The figure shows the reasoning result of individual 2 extracted from the case of city Z. In this scenario, residents were affected by public opinion and did not obtain the necessary information to judge the facts of the event, so they lacked the attribute of “knowledge increase”. With the addition of inferior individual resources with organizational advocacy capabilities, residents began to express their aspirations through practical actions. In order to show the evolution of this individual, a resident was first extracted as an individual with “hasLackOf” information, “hasAdd” organization skill, and “hasProduce” action attributes. After inference, the resident changed to “Fuzzy Citizens”, “Non-specialist Organization”, and triggered a group event. In the reasoning process of this example, three rules are involved in the reasoning operation:

1. Resident(?x) ^ Knowledge(?z) ^ hasLackOf(?x, ?z) -> FuzzyCitizens(?x);
2. FuzzyCitizens(?x) ^ Resources(?y) ^ hasAdd(?x, ?y) -> Non-specialistOrganization(?x);
3. Resident(?x) ^ Knowledge(?z) ^ hasLackOf(?x, ?z) ^ Resources(?y) ^ hasAdd(?x, ?y) ^ Requirement(?p) ^ hasProduce(?x, ?p) -> hasTrigger(?x, GroupIncident);

The above reasoning results show that when residents participate in the crisis event, if there are not enough factors to trigger the crisis transformation, such as “knowledge”, “resources”, etc., and they still have a certain demand on the outside world, the possibility of crisis event transformation to the positive direction is less in this scenario.

### 5.4. Risk Transformation Evaluation Structure

In order to clearly express the process of crisis transformation and evaluate the transformation effect, this article conducts transformation evaluation according to the dichotomy and grading method [80,81,82]. Based on Figure 7, with knowledge, resources and requirements as the criteria of dichotomous evaluation, the crisis transformation degree of output results is divided into four evaluation levels, forming a rapid crisis transformation evaluation structure, as shown in the Table 3.

In the crisis transformation evaluation structure, when the evolution path of a crisis event is forked at each node of the rule tree, it means that the direction and degree of risk transformation have changed. The crisis event evolves in a positive direction and the path with a higher degree of risk transformation is the optimal situation. This path has the characteristics of “KI”, “RJ” and “PR”. On the other side, the crisis event evolves in a negative direction which means the path holds a lower degree of risk transformation and heads to the worst situation. This path has the characteristics of “KS”, “RS” and “ER”. Under the three-level structure of the rule tree, a total of eight decision output paths are formed by using the dichotomy method [83]. Except for the above two optimal and worst scenarios, the remaining six decision-making output paths will have different impact on the final crisis transformation degree, due to the addition of different risk transformation factors in the upper two levels, and output different levels of risk transformation results.

In order to facilitate the reader’s intuitive analysis of the risk transformation process of crisis events and distinguish the different results of different scenario examples in the risk evolution process, the author sets an evaluation coefficient for the difference of each decision-making output path, which is four levels in total. A positive sign indicates the positive transformation direction and a negative sign indicates the negative transformation direction. The number of the evaluation coefficient itself does not have any meaning. Only when each decision output path is compared with each other does it represent a different degree of risk transformation [84].

## 6. Results and Discussion

Based on the setting of the above evaluation structure, 10 scenario individuals are sequentially established in the ontology. After starting the inference engine, 10 inference results will be obtained. According to the transformation path that matches the inference result, the evaluation coefficient is given, and the following results of the risk transformation reasoning of the MSW crisis event in Z city are obtained, as shown in Table 4. For example, the inference result of individual 1 is “FuzzyCitizens”, “IncompleteOrganization” and “hasPost FalseInformation”. According to the rule tree shown in Figure 7, the inference result of individual 1 conforms to the transformation path of “KS-RS-PR”. According to the evaluation system shown in Table 3, the evaluation coefficient of individual 1 is −3.

**Figure 11:**
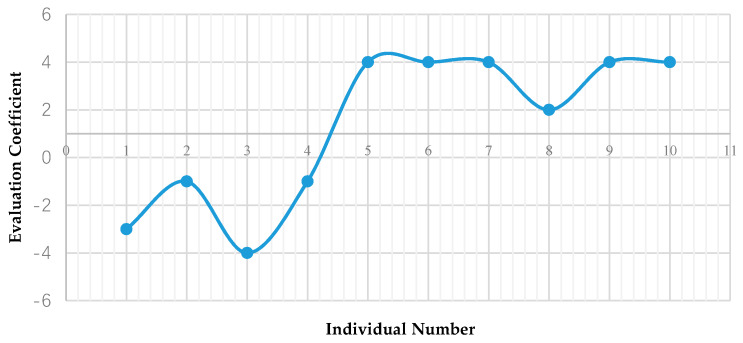
The risk transformation evaluation coefficient curve.

According to the risk transformation reasoning results in Table 4, the evaluation coefficients obtained after inference analysis of 10 scenario individuals are converted into a two-dimensional graph, which can more intuitively show the crisis transformation process and risk transformation degree of the Z city case. As shown in Figure 11, the horizontal axis represents the individual numbers of 10 scenarios, arranged in chronological order, and the vertical axis represents the evaluation coefficient obtained after regular reasoning.

The initial objective of the study was to integrate the existing case information and expert experience in the MSW crisis field to explore crisis transformation points and provide decision support for future risk response systems. The result of this case study indicates that the risk response method based on ontology reasoning proposed in this paper is effective in exploring the key risk transformation points in the evolution of the MSW crisis, and predicts the evolution trend and situation of the MSW crisis according to the key information points. The crisis transformation evaluation coefficient curve shows that the overall crisis transformation of the NIMBY event in Z city shows a positive development trend. The conclusion drawn from the curve trend in Figure 11 is consistent with the analysis results of most experts and scholars of the Z city case, and it is also consistent with the actual evolution of the Z city case. This shows that the ontology based on MSW crisis risk response proposed in this study is reasonable in rule reasoning.

The evaluation curve based on the inference results explores two key risk transformation points of individual 3 and individual 8. Individual 3 obtained the lowest risk transformation evaluation coefficient. In the scenario of Individual 3, residents who lacked professional knowledge and were not properly organized made excessive demands for negotiation with the government. The inference results show that, in this situation, residents are easily separated from the current official consultative organization. In the situation of poor risk transformation, controlling this point can restrain the continuous deterioration of the crisis event, so it is a key point. Individual 8 is in a late stage of better risk transformation, but compared with the neighboring individuals 7 and 9, the evaluation coefficient is still lower. As the trough of the overall rise of the curve, individual 8 indicates that the degree of risk transformation will still decline. Decision makers need to ensure that risk transformation is carried out efficiently and continuously, so this is a point that needs to be improved.

At present, China is in a critical period of social transformation. Social contradictions are deepening and diversifying, public crises are highly diffusive, and the consequences of an outbreak are serious [85]. Crises contain risks, and they also give birth to opportunities. Identifying points of opportunity from crisis situations and effectively transferring risks are new perspectives on crisis management [59]. Based on the above analysis of the research results, the following suggestions can be provided to decision makers. Firstly, the early stage of a crisis event is a critical period in completing the risk transformation. Policy makers need to discover and control key factors in time to prevent the event from deteriorating. Secondly, individual 3 shows that decision-makers formulating reasonable policies are the key factors in completing the risk transformation. Finally, individual 8 stated that even in the later stages it is necessary to be vigilant at all times and spread professional knowledge to the public through the official platform to prevent similar crises from happening again. 

## 7. Conclusions

This paper focused on the increasingly serious problems of the MSW crisis and explored the risk response methods based on ontology inference technology from the perspective of crisis transformation. Ontology and inference techniques are significant for the integration of information and the use of knowledge. The method proposed in this paper made full use of the existing case resources and empirical text by constructing the ontology model system and SWRL rule base to realize the mining of key risk points during the evolution of crisis events. A NIMBY crisis case study was discussed in order to highlight our proposed method. By doing so, we verified the reasoning results of the inference system. The results showed that this study can effectively explore key risk transformation points and analyze risk evolution trend and provide decision support for decision makers based on the information obtained by reasoning, which was helpful to in completing the risk response.

At present, there are few studies on MSW crisis management using ontology technology, and the research on crisis transformation has not formed a complete theoretical system. Although the risk response system regarding the MSW crisis, based on ontology reasoning, successfully realized the analysis of risk transformation and explored key factors of crisis events, there are a number of challenges associated with information acquisition and rule writing, since the quantity of information in the modern era surpasses our harnessing and capture capabilities. Accordingly, future work should involve innovative ways to support information acquisition, data mining, ontology application and logical reasoning. Furthermore, this method will be applied to more cases, so that the reasoning effect of the system will be improved by continuously increasing the amount of information and association of sample cases.

## Figures and Tables

**Table 1:** The relations in response strategy ontology model.

Classes	Object Properties	Classes
MSW crisis	*hasStrategy*	Response strategy set
Response strategy set	*hasApplyTo*	MSW crisis
	*hasResult*	Result
Subject	*isPartOf*	Response strategy set
	*use*	Resource
	*hasAction*	Action
Object	*isPartOf*	Response strategy set
Resource	*isPartOf*	Response strategy set
Action	*isPartOf*	Response strategy set
	*actionOn*	Object

**Table 2:** SWRL-based rule base.

Name	Body
1	Resident(?x) ^ Knowledge(?z) ^ hasIncrease(?x, ?z) -> ProfessionalCitizens(?x)
2	Resident(?x) ^ Knowledge(?z) ^ hasLackOf(?x, ?z) -> FuzzyCitizens(?x)
3	ProfessionalCitizens(?x) ^ Resources(?y) ^ hasAdd(?x, ?y) -> CompleteOrganization(?x)
4	ProfessionalCitizens(?x) ^ Resources(?y) ^ hasLackOf(?x, ?y) -> IncompleteOrganization(?x)
5	FuzzyCitizens(?x) ^ Resources(?y) ^ hasIncrease(?x, ?y) -> Non-specialistOrganization(?x)
6	FuzzyCitizens(?x) ^ Resources(?y) ^ hasLackOf(?x, ?y) -> WeakOrganization(?x)
7	Laws(?a) ^ Government(?q) ^ Resident(?x) ^ Knowledge(?y) ^ Resources(?z) ^ Requirement(?p) ^ hasIncrease(?x, ?y) ^ hasAdd(?x, ?z) ^ hasProduce(?x, ?p) -> hasEstablish(?q, ?a)
8	EnvironmentalDepartment(?a) ^ Government(?q) ^ Resident(?x) ^ Knowledge(?y) ^ Resources(?z) ^ Requirement(?p) ^ hasIncrease(?x, ?y) ^ hasAdd(?x, ?z) ^ hasProduce(?x, ?p) -> hasEstablish(?q, ?a)
9	SocialResponsibility(?a) ^ Enterprise(?q) ^ Resident(?x) ^ Knowledge(?y) ^ Resources(?z) ^ Requirement(?p) ^ hasIncrease(?x, ?y) ^ hasAdd(?x, ?z) ^ hasProduce(?x, ?p) -> hasUndertake(?q, ?a)
10	NegotiationChannel(?a) ^ Government(?q) ^ Resident(?x) ^ Knowledge(?y) ^ Resources(?z) ^ Requirement(?p) ^ hasIncrease(?x, ?y) ^ hasAdd(?x, ?z) ^ hasProduce(?x, ?p) -> hasEstablish(?q, ?a)
11	ENGO(?a) ^ Resident(?x) ^ Knowledge(?y) ^ Resources(?z) ^ Requirement(?p) ^ hasIncrease(?x, ?y) ^ hasAdd(?x, ?z) ^ hasProduce(?x, ?p) -> hasPromote(?x, ?a)
12	ConsensusRate(?a) ^ Government(?q) ^ Resident(?x) ^ Knowledge(?y) ^ Resources(?z) ^ Requirement(?p) ^ hasIncrease(?x, ?y) ^ hasAdd(?x, ?z) ^ hasExcessiveDemand(?x, ?p) -> hasReduce(?q, ?a)
13	FalseInformation(?a) ^ Resident(?x) ^ Knowledge(?z) ^ hasLackOf(?x, ?z) ^ Resources(?y) ^ hasLackOf(?x, ?y) ^ Requirement(?p) ^ hasProduce(?x, ?p) -> hasPost(?x, ?a)
14	GroupIncident(?a) ^ Resident(?x) ^ Knowledge(?z) ^ hasLackOf(?x, ?z) ^ Resources(?y) ^ hasAdd(?x, ?y) ^ Requirement(?p) ^ hasProduce(?x, ?p) -> hasTrigger(?x, ?a)
15	CompensationStandards(?a) ^ Resident(?x) ^ Knowledge(?z) ^ hasLackOf(?x, ?z) ^ Resources(?y) ^ hasAdd(?x, ?y) ^ Requirement(?p) ^ hasExcessiveDemand(?x, ?p) -> hasImprove(?x, ?a)
16	ConsultativeOrganization(?a) ^ Resident(?x) ^ Knowledge(?z) ^ hasLackOf(?x, ?z) ^ Resources(?y) ^ hasLackOf(?x, ?y) ^ Requirement(?p) ^ hasExcessiveDemand(?x, ?p) -> hasDetach(?x, ?a)
17	PublicOpinion(?a) ^ Resident(?x) ^ Knowledge(?z) ^ hasLackOf(?x, ?z) ^ Resources(?y) ^ hasAdd(?x, ?y) ^ Requirement(?p) ^ hasProduce(?x, ?p) -> hasTrigger(?x, ?a)
18	InformationTransparency(?a) ^ Government(?q) ^ Resident(?x) ^ Knowledge(?z) ^ hasLackOf(?x, ?z) ^ Resources(?y) ^ hasLackOf(?x, ?y) ^ Requirement(?p) ^ hasExcessiveDemand(?x, ?p) -> hasImprove(?q, ?a)
19	ConsensusRate(?a) ^ Resident(?x) ^ Knowledge(?y) ^ Resources(?z) ^ Requirement(?p) ^ hasIncrease(?x, ?y) ^ hasAdd(?x, ?z) ^ hasProduce(?x, ?p) -> hasImprove(?x, ?a)
20	ProfessionalKnowledge(?a) ^ Resident(?x) ^ Knowledge(?y) ^ Resources(?z) ^ Requirement(?p) ^ hasIncrease(?x, ?y) ^ hasLackOf(?x, ?z) ^ hasProduce(?x, ?p) -> hasIncrease(?x, ?a)
21	Participation(?a) ^ Resident(?x) ^ Knowledge(?y) ^ Resources(?z) ^ Requirement(?p) ^ hasIncrease(?x, ?y) ^ hasAdd(?x, ?z) ^ hasProduce(?x, ?p) -> hasImprove(?x, ?a)

**Table 3:** The rapid risk transformation evaluation system.

Transformation Path	Evaluation Coefficient
KI-RJ-PR	+4
KI-RJ-ER	+3
KI-RS-PR	+2
KI-RS-ER	+1
KS-RJ-PR	−1
KS-RJ-ER	−2
KS-RS-PR	−3
KS-RS-ER	−4

**Table 4:** The inference result of individual in the case in Z city.

Individual		Participating SWRL Rules	Inference Results	Evaluation Coefficient
1	Resident1 hasLackOf information/ hasLackOf organizationskill/ hasProduce communication/	2 4 13	FuzzyCitizens/ IncompleteOrganization/ Resident hasPost FalseInformation/	−3
2	Resident2 hasLackOf information/ hasAdd organizationskill/ hasProduce action	2 3 14	FuzzyCitizens/ CompleteOrganization/ Resident hasTrigger GroupIncident/	−1
3	Resident3 hasLackOf information/ hasLackOf socialstatus/ hasExcessiveDemand negotiation	2 6 16	FuzzyCitizens/ WeakOrganization/ Resident hasDetach ConsultativeOrganization/	−4
4	Resident4 hasLackOf statute/ hasAdd organizationskill/ hasProduce negotiation	2 3 10	FuzzyCitizens/ CompleteOrganization/ Government hasEstablish NegotiationChannel/	−1
5	Resident5 hasAdd statute/ hasAdd socialstatus/ hasProduce regulation	1 3 7	ProfessionalCitizens/ CompleteOrganization/ Government hasEstablish Laws/	+4
6	Resident6 hasAdd EnvironmentalAwareness/ hasAdd OrganizationSkills/ hasProduce Action	1 3 19	ProfessionalCitizens/ CompleteOrganization/ Government hasImprove ConsensusRate/	+4
7	Resident7 hasAdd EnvironmentalAwareness/ hasAdd OrganizationSkills/ hasProduce information	1 3 20	ProfessionalCitizens/ CompleteOrganization/ Resident hasincrease ProfessionalKnowledge/	+4
8	Resident8 hasAdd Professional knowledge/ hasALackOfOrganizationSkills/ hasProduce information	1 4 11	ProfessionalCitizens/ IncompleteOrganization/ Resident hasPromote ENGO/	+2
9	Resident9 hasAdd ProfessionalKnowledge/ hasAdd OrganizationSkills/ hasProduce Action	1 3 21	ProfessionalCitizens/ CompleteOrganization/ Resident hasImprove Participation/	+4
10	Resident10 hasAdd EnvironmentalAwareness/ hasAdd Operating platform/ hasProduceAction	1 3 9	ProfessionalCitizens/ CompleteOrganization/ EnterprisehasUndertake SocialResponsibility	+4

## Appendix A. Case Scenario Description

**Table A1:** Case Scenario Description in Time Node.

Date	Scenario Description
21 June 2016	Some people in Z city published the news about Z city will build a waste incineration plant on local Internet forums and other platforms, which aroused public attention.
24 June 2016	Public opinion is gradually heating up, and some people have launched a boycott initiative on social platforms. On the 25th, the incident broke out and the conflict reached a deadlock. Some people took to the streets for violent protests, boycotted the construction of Z municipal domestic waste incineration power station project, and the government sent out armed police.
25 June 2016	A video of police-civilian conflict was widely spread on social platforms, and the incident continues to ferment. At seven o’clock in the evening, the city lost power. In order to alleviate the public’s mood and answer the public’s doubts, the government of Z city held a news conference on the municipal solid waste incineration power generation project, and important government officials also spoke intensively on the same day to explain the necessity of the project, to show their support.
26 November 2016	After the outbreak of the incident, the government made a deep reflection and made positive rectification. Then Z city circular economy industrial park was established. Six working groups, including the comprehensive coordination group, the publicity and public opinion guidance group, the process optimization group, the engineering construction group, the environmental remediation working group, and stability maintenance group, quickly started the work.
25 December 2016	Through participating in the political and civil exchange meeting held by the government, the representative of the citizens expressed suggestions of the municipal solid waste incineration power generation project.
13 January 2017	Z city organized some villagers to visit and investigate the built municipal solid waste incineration power plant in W city. The plant area is covered with green trees and lawns, which are neither smelly nor smoky, and are praised by the villagers.
5 April 2017	Z City has carried out scientific and educational publicity activities on municipal solid waste incineration. Government officials form working groups to popularize scientific knowledge in neighborhood communities.
13 April 2017	A government official of Z city published an article on the social platform explaining the necessity and safety of the waste incineration project, which was forwarded by many people and greatly promoted the waste incineration power generation project.
3 May 2017	With the support rate of 99%, the upgraded version of the municipal solid waste incineration power generation project in Z city was restarted at the original site. On May 25th, the training meeting for volunteer supervisors of the waste incineration power generation project was held.
15 April 2018	The project was put into trial operation. The huge LCD screen on the entire wall of the central control room of the trial operation of the waste incineration plant displays the entire process of waste entering, storage, incineration, slag discharge, and various flue gas emission indicators in real time. This truly realizes the openness and transparency of information.

## Appendix B. The Individual Extraction

**Table A2:** The individual extraction.

Individual Number	Time	Situational Interpretation	Object Property
1	21 June 2016	In the case of insufficient information and no professionals or professional organizations to explain and guide, residents have demanded the outside world for the purpose of protecting their own interests and have the behavior of issuing speeches.	hasLackOf information/ hasLackOf organizationskill/ hasProduce communication/
2	24 June 2016	In the case of incomplete information, residents were guided by people with organizational skills. In order to express their opposition to the project under construction, they participated in discussions of topics and participated in resistance activities.	hasLackOf information/ hasAdd organizationskill/ hasProduce action/
3	25 June 2016	Under the influence of incomplete information, residents have a large resistance, and in the absence of an organized and good social status to talk to the government, they asked the government to give an explanation of the incident.	hasLackOf information/ hasLackOf socialstatus/ hasExcessiveDemand negotiation/
4	26 November 2016	Residents who lack relevant legal knowledge and communication channels express their demands to the government by participating in mass incidents and require the establishment of negotiation channels with the government.	hasLackOf statute/ hasAdd organizationskill/ hasProduce negotiation/
5	25 December 2016	Residents with knowledge of relevant laws and environmental protection, by participating in the government-civilian exchange meeting, make requirements on government work by making relevant suggestions.	hasAdd statute/ hasAdd socialstatus/ hasProduce regulation/
6	13 January 2017	Residents who raise awareness of environmental protection accept the organization of relevant government departments, and actually participate in the science popularization activities of waste incineration projects for the purpose of solving problems.	hasAdd EnvironmentalAwareness/ hasAdd OrganizationSkills / hasProduce Action/
7	5 April 2017	Residents who lack relevant professional knowledge but are environmentally conscious will accept the popularization of organized scientific and educational knowledge of relevant government departments.	hasAdd EnvironmentalAwareness/ hasAddOrganizationSkills/ hasProduce information/
8	13 April 2017	Residents with relevant professional knowledge participate in the propaganda work of the industrial park in an individual form to guide the sound development of the industrial park.	hasAdd Professional knowledge/ hasLackOf OrganizationSkills/ hasProduce information/
9	3 May 2017	After eliminating the prejudice against the waste incineration project and improving the recognition of the project, residents with professional knowledge participate in the environmental impact assessment supervision of the waste incineration project under the organization of the government or enterprises.	hasAdd ProfessionalKnowledge/ hasAdd OrganizationSkills/ hasProduce Action/
10	15 April 2018	After the project is completed, in accordance with the requirements of residents with high environmental awareness, the waste incineration plant needs to make information public, so that residents can monitor the operation of the waste incineration plant in real time.	hasAdd EnvironmentalAwareness/ hasAdd Operating platform/ hasProduceAction/
